# Supplementary figures and images for: Meiosis-specific prophase-like pathway controls cleavage-independent release of cohesin by Wapl phosphorylation
Source: PLoS Genet. 2019 Jan 3;15(1):e1007851. doi: 10.1371/journal.pgen.1007851 (PMC6317811; doi:10.1371/journal.pgen.1007851)

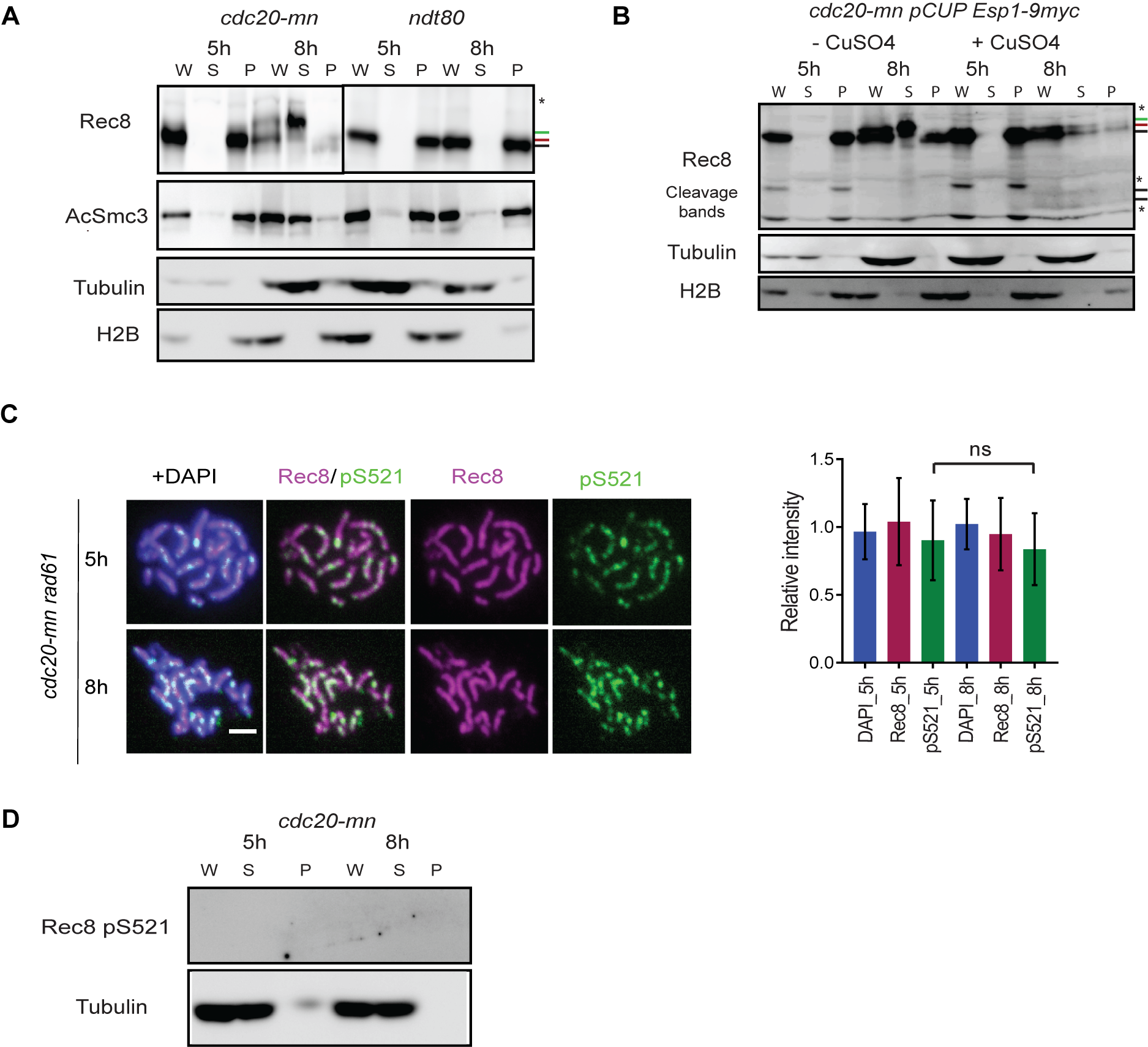

Supplement: S1 Fig — A: Chromatin fractionation assay was carried out using CDC20-mn (KSY642/643) and ndt80 (KSY467/468) mutant cells. Western blotting was performed for whole cell extracts (W), soluble fractions (S) and chromatin-bound fraction (P). Rec8 (top) and acetyl-Smc3 (second) were probed together with tubulin (third) and Histone 2B (H2B; bottom) as controls for soluble and chromatin-bound proteins, respectively. B: Chromatin fractionation assay of CDC20-mn pCUP-Esp1-9myc (KSY1009/1010) cells without and with overexpression of Esp1 was carried out as shown in (A). C: Localization of Rec8 (red) and Rec8-pS521 (phospho-S521; green) was analyzed in cdc20-mn rad61 (KSY637/638) cells at 5 and 8 h. Total Rec8, Rec8-pS521, and DAPI signal intensity was studied as in Fig 1C and shown in bottom. Error bars show the S.D. (n = 3). D: Western blotting of Rec8 pS521 in CDC20-mn (KSY642/643) was done with tubulin as a control. (TIF) [file pgen.1007851.s001.tif]

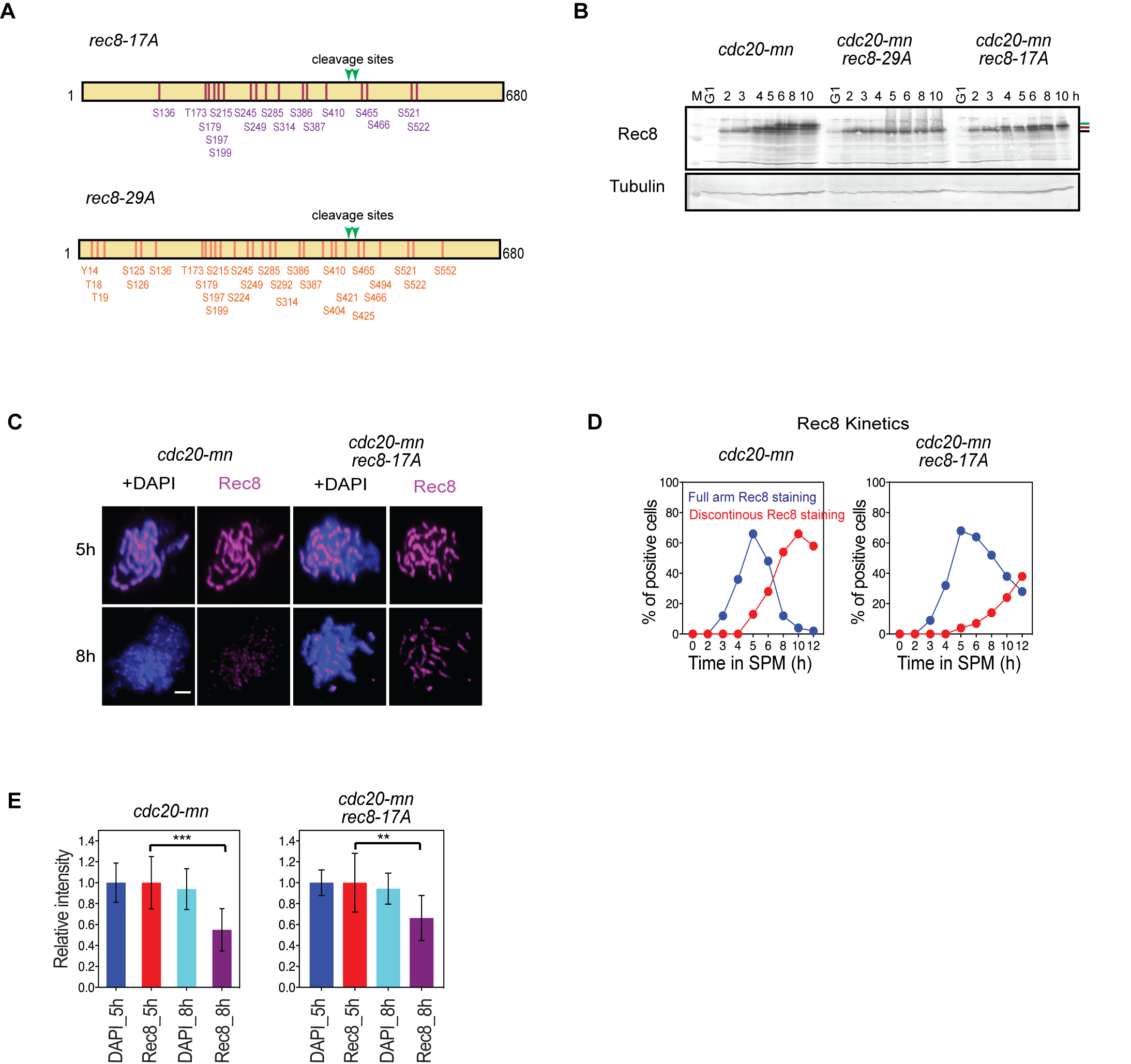

Supplement: S2 Fig — A: Schematic drawing of Rec8-17A and Rec8-29A mutant proteins. Mutated amino acid residues are shown in red. B: Western blotting analysis of Rec8 and tubulin was carried out using CDC20-mn (KSY642/643), CDC20-mn rec8-29A (KSY866/867) and CDC20-mn rec8-17A (KSY812/813) cells strain as described (A). Phosphorylated species of Rec8 and tubulin. Representative images are shown. C: Localization of Rec8 (red) on chromosome spreads was analyzed for CDC20-mn (KSY642/643) and CDC20-mn rec8-17A (KSY812/813) cells. Representative image with or without DAPI (blue) dye is shown. The bar indicates 2μm. D: Kinetics of Rec8 staining classes in (C) was analyzed as in Fig 1B. A minimum 100 cells were analyzed at each time point. E: Quantified total Rec8 and DAPI signal intensity was measured. A minimum 30 nuclei were quantified in each representative time points. Error bars show the S.D. (n = 3). (TIF) [file pgen.1007851.s002.tif]

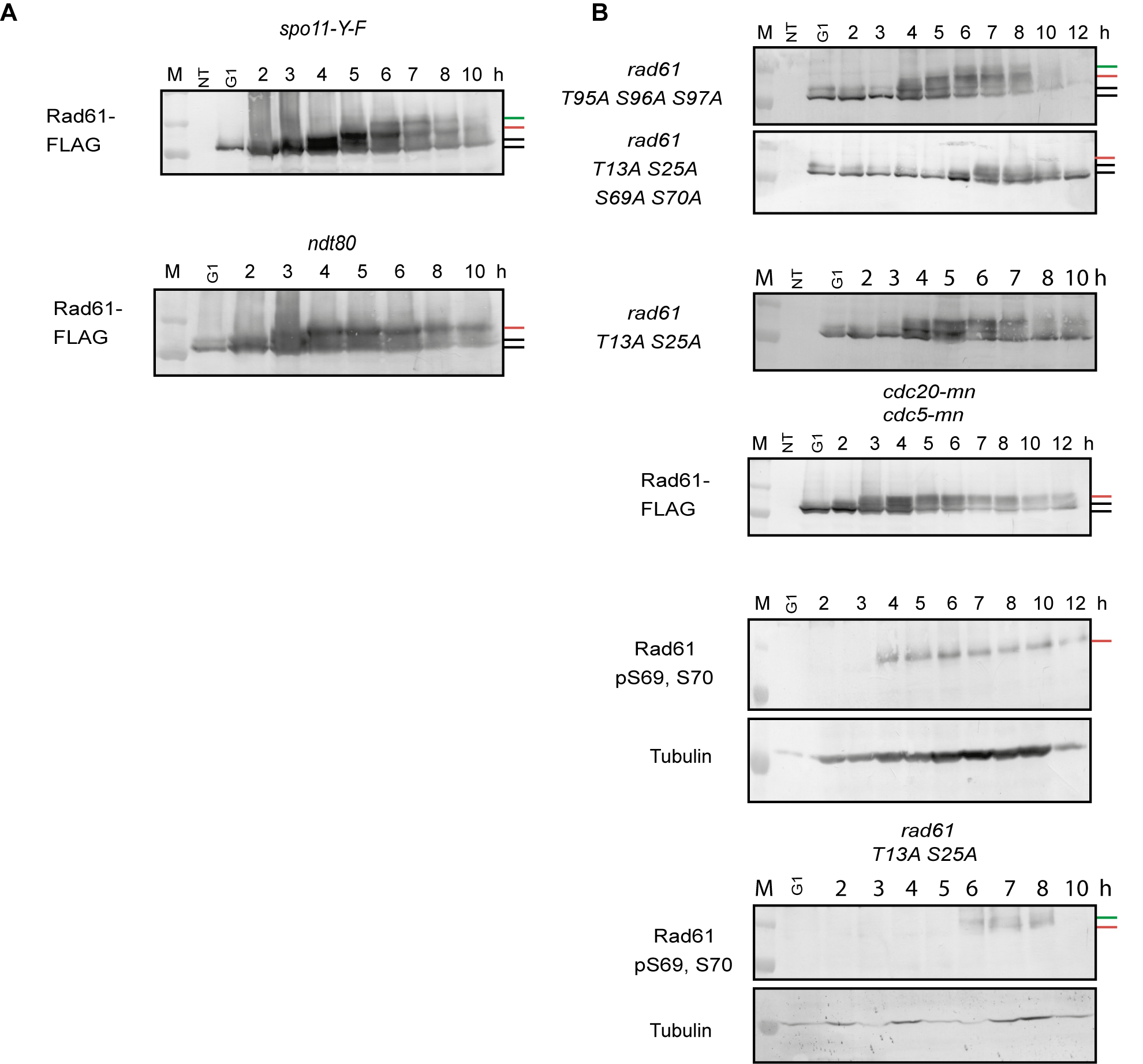

Supplement: S3 Fig — A: The western blotting analysis was carried out for Rad61-Flag in RAD61-FLAG (KSY440/441), rad61-S69AS70A-FLAG (KSY754/757) and rad61-7A-FLAG (KSY753/755) strains. B: Bands shits of Rad61 in ndt80 RAD61-FLAG (KSY467/468) and spo11-Y135F RAD61-FLAG (KSY474/475) cells were analyzed as shown in (A). (TIF) [file pgen.1007851.s003.tif]

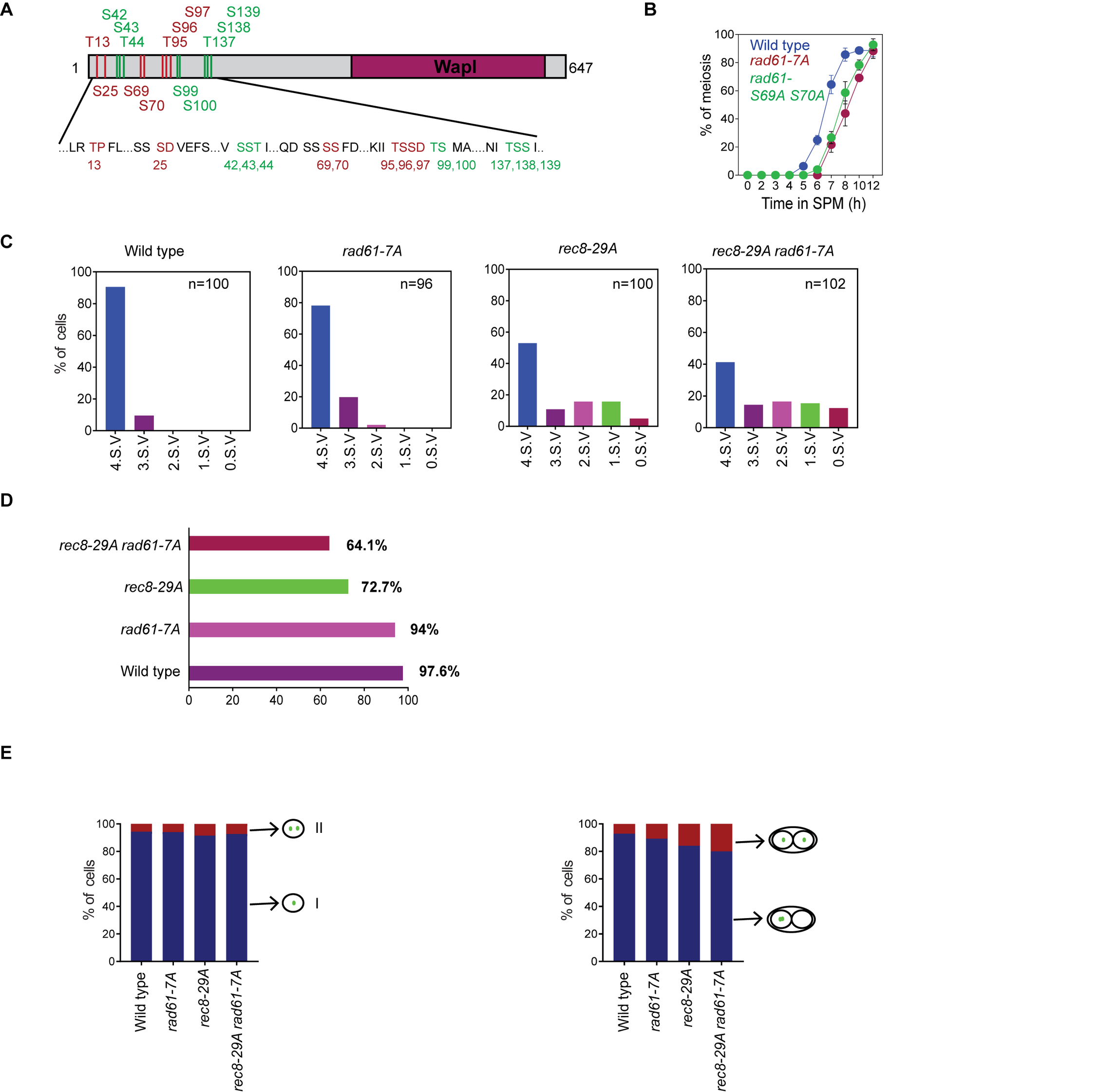

Supplement: S4 Fig — A: Schematic drawing of Rad61 with putative DDK-dependent (red) and PLK-dependent phosphorylation sites (green). Conserved “WAPL” domain is shown in a box. B: Kinetics of the entry into meiosis I in wild-type (MSY832/833) and rad61-7A (KSY753/755) cells was analyzed by DAPI counting. A cell with 2, 3, and 4 DAPI bodies was counted. At each time point, more than 100 cells were examined. C: Distribution of viable spores per tetrad in various strains was measured and shown. Spores were incubated after dissection at 30°C for 3 days. Each bar indicates the percentage of classes with 4, 3, 2, 1 and 0 viable spores per tetrad. Spore viability and the total number of dissected tetrads (parentheses) are also shown. Wild type (MSY832/833), rad61-7A (KSY753/755), rec8-29A (KSY814/815), rec8-29A rad61-7A (KSY982/983) cells. D: Percentage of viable spores in various strains was shown in graph. Wild type (MSY832/833), rad61-7A (KSY753/755), rec8-29A (KSY814/815), rec8-29A rad61-7A (KSY982/983) cells. E: Sister chromatid cohesion and segregation of homologous chromosome were analyzed. A cell heterozygous for CEN4-GFP locus was used. At least more than 50 cells with single and two DAPI bodies in a cell were examined for the number of CEN4-GFP spot at 4, 5, and 6 h. For sister chromatid cohesion assay (left graph), the number of a cell containing single DAPI body with either 1 or 2 GFP spots was counted. For segregation assay of homologous chromosomes at meiosis I (right graph), a cell containing two DAPI bodies was examined for either both two DAPI bodies contained 1 GFP spot or one of two DAPI bodies contained 1 or 2 spots. Wild type (MSY833/KSY216), rad61-7A (KSY653/1089), rec8-29A (KSY814/1086), rec8-29A rad61-7A (KSY982/1091) cells. (TIF) [file pgen.1007851.s004.tif]
