# Supplementary material for: Meiosis-specific prophase-like pathway controls cleavage-independent release of cohesin by Wapl phosphorylation
Source: PLoS Genet. 2019 Jan 3;15(1):e1007851. doi: 10.1371/journal.pgen.1007851 (PMC6317811; doi:10.1371/journal.pgen.1007851)
Supplement: S1 Table — The strain used in this study and its genotype. (DOCX) [file pgen.1007851.s005.docx]

**Table S1: Strain list**

**Strain Name** **Genotype**

| MSY832 | *MAT α, ho::LYS2, ura3, leu2::hisG, trp1::hisG, lys2* |
| --- | --- |
| MSY833 | *MAT a, ho::LYS2, ura3, leu2::hisG, trp1::hisG, lys2* |
| KSY440 | MSY 833 with *RAD61-3FLAG ::KanMX6* |
| KSY441 | MSY 832 with *RAD61-3FLAG ::KanMX6* |
| KSY434 | MSY 833 with *RAD61-3FLAG ::KanMX6, cdc5::pCLB2-CDC5::KanMX6* |
| KSY435 | MSY 832 with *RAD61-3FLAG ::KanMX6, cdc5::pCLB2-CDC5::KanMX6* |
| KSY467 | MSY 833 with *RAD61-3FLAG ::KanMX6, ndt80::LEU2* |
| KSY468 | MSY 832 with *RAD61-3FLAG ::KanMX6, ndt80::LEU2* |
| KSY474 | MSY 833 with *RAD61-3FLAG ::KanMX6, spo11-Y135F::KanMX6* |
| KSY475 | MSY 832 with *RAD61-3FLAG ::KanMX6, spo11-Y135F::KanMX6* |
| KSY627 | MSY 833 with *RAD61-3FLAG ::KanMX6, rec8::KanMX6* |
| KSY628 | MSY 832 with *RAD61-3FLAG ::KanMX6, rec8::KanMX6* |
| KSY637 | MSY 833 with *cdc20::pCLB2-CDC20::KanMX6, rad61::KanMX6* |
| KSY638 | MSY 832 with *cdc20::pCLB2-CDC20::KanMX6, rad61::KanMX6* |
| KSY642 | MSY 833 with *cdc20::pCLB2-CDC20::KanMX6* |
| KSY643 | MSY 832 with *cdc20::pCLB2-CDC20::KanMX6* |
| KSY659 | MSY 833 with *cdc20::pCLB2-CDC20::KanMX6, cdc5::pCLB2-CDC5::KanMX6* |
| KSY660 | MSY 833 with *cdc20::pCLB2-CDC20::KanMX6, cdc5::pCLB2-CDC5::KanMX6* |
| KSY753 | MSY 833 with *rad61-7A-3FLAG ::KanMX6* |
| KSY755 | MSY 832 with *rad61-7A-3FLAG::KanMX6* |
| KSY882 | MSY 833 with *Pcdc5-CDC5::pGAL1-CDC5N209A::HphMX4,*  *pGPD1-GAL4.ER::URA3, ndt80::LEU2* |
| KSY883 | MSY 832 with *Pcdc5-CDC5::pGAL1-CDC5N209A::HphMX4,*  *pGPD1-GAL4.ER::URA3, ndt80::LEU2* |
| KSY887 | MSY 833 with *Pcdc5-CDC5::pGAL1-CDC5::HphMX4,*  *pGPD1-GAL4.ER::URA3, ndt80::LEU2* |
| KSY888 | MSY 832 with *Pcdc5-CDC5::pGAL1-CDC5::HphMX4,*  *pGPD1-GAL4.ER::URA3, ndt80::LEU2* |
| KSY866 | MSY 833 with *rec8::KanMX::rec8-29A::LEU2, cdc20::pCLB2-CDC20::KanMX6* |
| KSY867 | MSY 832 with *rec8::KanMX::rec8-29A::LEU2, cdc20::pCLB2-CDC20::KanMX6* |
| KSY978 | MSY 833 with *RAD61-3FLAG ::KanMX6, cdc7-as3-myc* |
| KSY979 | MSY 832 with *RAD61-3FLAG::KanMX6, cdc7-as3-myc* |
| KSY982 | MSY 833 with *rec8::KanMX::rec8-29A::LEU2, rad61-7A-3FLAG ::KanMX6* |
| KSY983 | MSY 832 with *rec8::KanMX::rec8-29A::LEU2, rad61-7A-3FLAG ::KanMX6* |
| KSY991 | MSY832 with *leu2::LacI-GFP::Clonat, trp1::226XlacO::KanMX4,*  *telIV:: 226XlacO::KanMX4, cdc20::pCLB2-CDC20::KanMX6* |
| KSY1043 | MSY 833 with *rec8::KanMX::rec8-29A::LEU2, rad61-7A-3FLAG ::KanMX6,*  *cdc20::pCLB2-CDC20::KanMX6* |
| KSY1044 | MSY 832 with *rec8::KanMX::rec8-29A::LEU2, rad61-7A-3FLAG ::KanMX6,*  *cdc20::pCLB2-CDC20::KanMX6* |
| KSY597 | Mat 833/832 with *REC8N-HA3(ER)-LEU2::REC8::KanMX4* |
| KSY1009 | MSY 833 with *cdc20::pCLB2-CDC20::KanMX6, pCUP1-KanMX-ESP1* |
| KSY1010 | MSY 832 with *cdc20::pCLB2-CDC20::KanMX6, pCUP1-KanMX-ESP1* |
| KSY754 | MSY 833 with *rad61-69A,70A-3FLAG ::KanMX6* |
| KSY757 | MSY 832 with r*ad61-69A,70A-3FLAG::KanMX6* |
| KSY989 | MSY832 with *leu2::LacI-GFP::Clonat, trp1::226XlacO::KanMX4,*  *telIV:: 226XlacO::KanMX4, cdc20::pCLB2-CDC20::KanMX6,*  *cdc5::pCLB2-CDC5:KanMX6* |
| KSY445 | MSY832 with *leu2::LacI-GFP::Clonat, trp1::226XlacO::KanMX4,*  *telIV:: 226XlacO::KanMX4, ndt80::LEU2* |
| KSY814 | MSY 833 with *rec8::KanMX::rec8-29A::LEU2* |
| KSY815 | MSY 832 with *rec8::KanMX::rec8-29A::LEU2* |
| KYS653 | MSY 833 with *cdc20::pCLB2-CDC20::KanMX6, rad61-7A-3FLAG::KanMX6* |
| KYS654 | MSY 832 with *cdc20::pCLB2-CDC20::KanMX6, rad61-7A-3FLAG::KanMX6* |
| KYS478 | MSY 833 with *cdc20::pCLB2-CDC20::KanMX6, RAD61-3FLAG::KanMX6* |
| KYS479 | MSY 832 with *cdc20::pCLB2-CDC20::KanMX6, RAD61-3FLAG::KanMX6* |
| KYS1086 | MSY 832 with *rec8-29A* KanMX6  *CenIV trp1:226lacO::KamMX4, leu2::LacI-GFP:Clonat* |
| KYS1089 | MSY 832 with *rad61-7A* KanMX6  *CenIV trp1:226lacO::KamMX4, leu2::LacI-GFP:Clona*t |
| KYS1091 | MSY 832 with *rec8-29A KanMX6 rad61-7A KanMX6*  *CenIV trp1:226lacO::KamMX4, leu2::LacI-GFP:Clonat* |
| KYS1093 | MSY 833 with *rec8 KanMX6 cdc5::pCLB2-CDC5::KanMX6*  *RAD61-3FLAG::KanMX6* |
| KYS1092 | MSY 832 with *rec8 KanMX6 cdc5::pCLB2-CDC5::KanMX6*  *RAD61-3FLAG::KanMX6* |
